# Deciphering Cell-Cell Communication in Abdominal Aortic Aneurysm From Single-Cell RNA Transcriptomic Data

**DOI:** 10.3389/fcvm.2022.831789

**Published:** 2022-02-04

**Authors:** Huan Yang, Elise DeRoo, Ting Zhou, Bo Liu

**Affiliations:** ^1^Department of Surgery, School of Medicine and Public Health, University of Wisconsin-Madison, Madison, WI, United States; ^2^Department of Cellular and Regenerative Biology, School of Medicine and Public Health, University of Wisconsin-Madison, Madison, WI, United States

**Keywords:** abdominal aortic aneurysm, cell-cell communication, single-cell RNA sequencing, thrombospondin, animal models

## Abstract

Cell-cell communication coordinates cellular differentiation, tissue homeostasis, and immune responses in states of health and disease. In abdominal aortic aneurysm (AAA), a relatively common and potentially life-threatening vascular disease, intercellular communications between multiple cell types are not fully understood. In this study, we analyzed published single-cell RNA sequencing (scRNA-seq) datasets generated from the murine CaCl_2_ model, perivascular elastase model, Angiotensin II model, and human AAA using bioinformatic approaches. We inferred the intercellular communication network in each experimental AAA model and human AAA and predicted commonly altered signaling pathways, paying particular attention to thrombospondin (THBS) signaling between different cell populations. Together, our analysis inferred intercellular signaling in AAA based on single-cell transcriptomics. This work provides important insight into cell-cell communications in AAA and has laid the groundwork for future experimental investigations that can elucidate the cell signaling pathways driving AAA.

## Introduction

Abdominal aortic aneurysm (AAA), defined as a focal dilation of the abdominal aorta beyond 50% of its normal diameter, is a common and potentially lethal aortic disease (1). Decades of basic and clinical research have revealed multiple molecular processes that underlie the development and growth of AAAs, including infiltration of immune cells, degeneration of extracellular matrix (ECM), and depletion of medial smooth muscle cells (SMCs) (2). Experimental data also implicate the importance of intercellular communication between inflammatory cells and SMCs during aneurysm development (3). Various anti-inflammatory strategies that were found to prevent aneurysm formation in mice were shown to reduce SMC death and preserve the contractile phenotype in the aortic wall (4, 5). Reciprocally, inhibiting cell death in aneurysm models has been shown to reduce intra-aortic accumulation of inflammatory cells (6, 7). Despite these early insights, a comprehensive understanding of communication patterns between different cell populations in healthy and aneurysmal aorta remains elusive.

Single-cell RNA sequencing (scRNA-seq) is a powerful research tool that has been recently employed by multiple groups to investigate transcriptomic profiles of human and experimental aortic aneurysm tissue at single-cell resolution (3, 8–11). The large data sets produced by published scRNA-seq studies confirmed the involvement of multiple cell types and subtypes in aneurysm pathophysiology. In addition, the published scRNA-seq data contained information on gene expression of ligands, receptors, and cofactors that could be used to analyze cell-cell communication status in the tissues (12, 13). CellChat is an analytic tool developed by Jin and colleagues that quantitatively deduces intercellular communication networks from scRNA-seq data (14). In this study, we applied CellChat to our scRNA-seq dataset as well as other published datasets generated from analyzing murine and human AAA tissues. Our data inferred the intercellular communication status of healthy and diseased aortas, and predicted potential signaling pathways altered by AAA in each model.

## Materials and Methods

CaCl_2_-, elastase-, or Angiotensin II (Ang II) induced mouse experimental AAA as well as human AAA scRNA-seq datasets were downloaded from the NCBI GEO data repository (GSE164678, GSE152583, GSE118237, and GSE166676). Data preprocessing, normalization, scaling, and cell clustering were performed with Seurat package (version 4.0.3) in R (version 4.1.1) environment (15). Cell populations were determined using the marker genes in the original studies (3, 9–11). Red blood cells were excluded for cell-cell interaction analysis. Seurat preprocessed data was then subjected to CellChat package (version 1.1.3) to infer, analyze, and visualize cell-cell communication (14). The ligand-receptor interaction database was included in the package. Conserved and context-specific signaling pathways identified by CellChat were subjected to EVenn to generate Venn diagrams or Venn networks for the visualization for set relationships (16).

### Statistics

Statistical analysis was performed within the CellChat package. Interaction strength represents ligand-receptor mediated intercellular communication probability, quantified by the law of mass action. Incoming (or outgoing) interaction strength is the communication probabilities of the incoming (or outgoing) signaling to (or from) a cell population. The overall information flow for a given signaling pathway is the sum of communication probability among all pairs of cell groups in the inferred network (14).

## Results

### Cell-Cell Communication in Murine CaCl_2_ Model

CellChat is an R toolkit that includes a database comprising 2,021 validated mouse molecular interactions or 1,939 human molecular interactions between signaling ligands, receptors, and their cofactors (14). The communication probability of a specific signaling pathway (such as COLLAGEN signaling) was the sum of the communication probability of each ligand-receptor pair of that specific signaling pathway. We evaluated cell-cell communication patterns in the murine CaCl_2_ model by applying CellChat to the scRNA-seq dataset published by our lab (GSE164678). In that study, we perivascularly treated the infrarenal abdominal aortas of C57BL/6J mice with 0.5 M CaCl_2_ (AAA group) or NaCl (sham group). Aortas were collected 4 days after AAA induction (10) to capture acute transcriptional responses within the aortic wall. This CaCl_2_ scRNA-seq dataset contains 3,896 cells in total, including 2,537 cells from the sham group and 1,359 cells from the AAA group. Cells were clustered into 12 populations, including two fibroblast (Fib), two smooth muscle cell (SMC), and three macrophage (Maph) populations, as well as several other cell types such as endothelial cell (EC), neutrophil (Neutro), dendritic cell (DC), T and natural killer cell (T/NK), and B cell (Supplementary Figure 1A).

**Figure 1:**
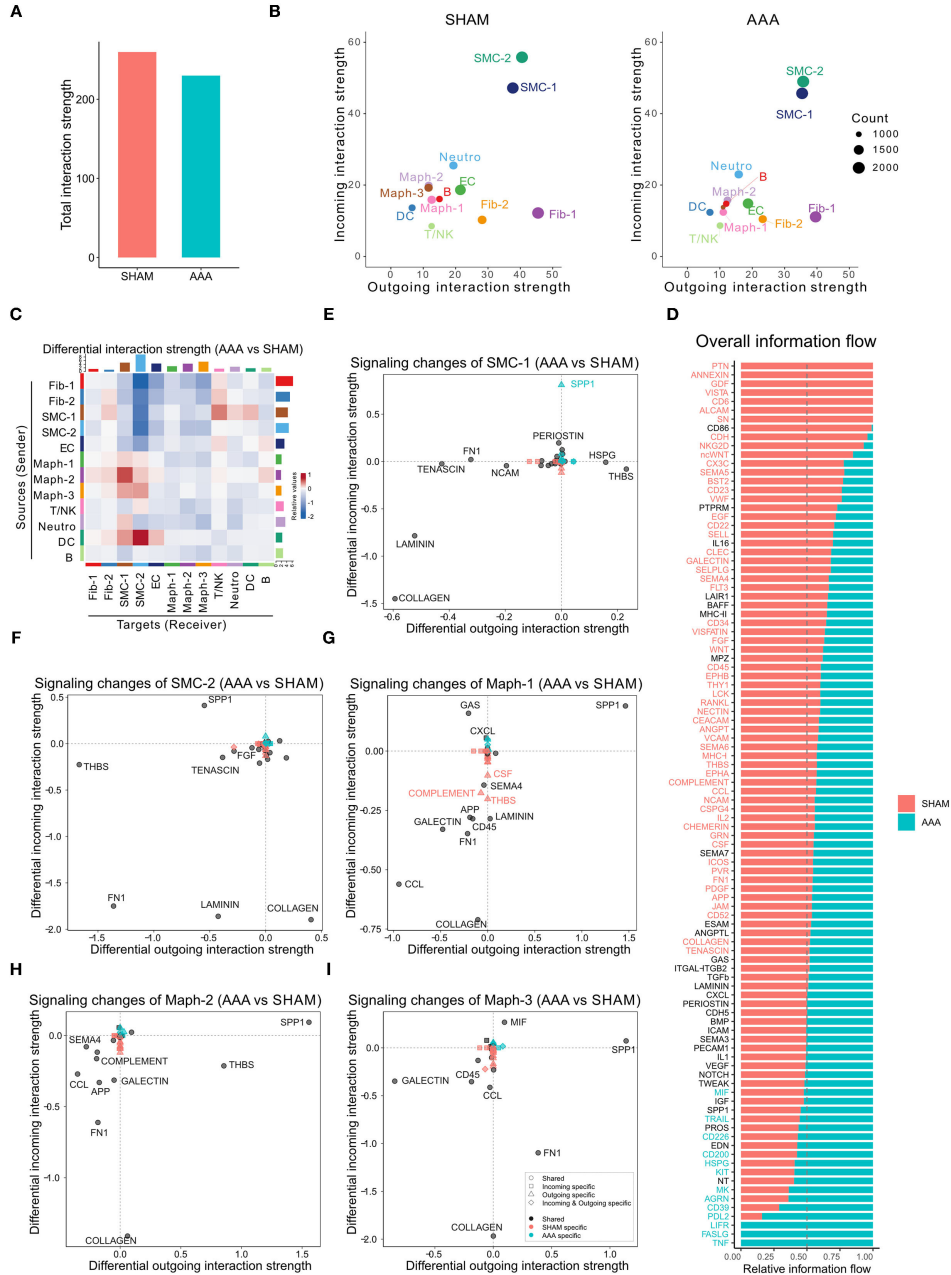
Inferred intercellular communication network in the murine CaCl_2_ model. **(A)** Total interaction strength in sham (NaCl treated) and AAA (CaCl_2_ treated) groups. **(B)** Scatter plot of incoming and outgoing interaction strength of each cell population in sham and AAA groups. **(C)** Heatmap of differential interaction strength in AAA group compared to sham group. The top colored bar plot represents the sum of column of values displayed in the heatmap (incoming signaling). The right colored bar plot represents the sum of row of values (outgoing signaling). In the heatmap, red (or blue) represents increased (or decreased) signaling in AAA compared to sham group. Relative value = the interaction strength from source to target in AAA group – the interaction strength from source to target in sham group. **(D)** Overall information flow of each signaling pathway in sham and AAA groups. Relative information flow is the ratio of the communication probability of a certain group (sham or AAA group) relative to sham and AAA combined. **(E–I)** Signaling changes of SMC-1 **(E)**, SMC-2 **(F)**, Maph-1 **(G)**, Maph-2 **(H)**, Maph-3 **(I)** in AAA compared to sham group.

CellChat analysis of the CaCl_2_ dataset revealed 8,799 total ligand-receptor interactions in the sham group and 8,601 interactions in the AAA group (Supplementary Figure 1B). The strength of a given ligand-receptor interaction is quantified by a probability value. This probability value is modeled by the law of mass action based on the average expression value of a ligand by one cell group and the expression value of a corresponding receptor in another cell group, as well as the cofactors of the ligand or receptor (14). On post-surgery day 4, the total interaction strength of the AAA group was moderately lower than that of the sham group (Figure 1A). When comparing the outgoing and incoming signals of each cell population in sham and AAA tissues, we found that SMCs and Fib-1 were the major signaling sources, and that SMCs also functioned as the major signaling target in both tissues (Figure 1B). Compared to the sham group, AAA induction increased signals sent from Maph-2 to SMC-1 and from DC to SMC-2, and decreased signals from fibroblasts (Fib-1 and Fib-2) and SMCs (SMC-1 and SMC-2) to SMC-2 (Figure 1C).

To identify the conserved and context-specific signaling pathways induced by AAA, we compared the overall information flow for each signaling pathway, which was defined by the sum of communication probability among all cell populations in each condition (14). As shown in Figure 1D, the majority of signaling pathways were found in both sham and AAA groups. There were seven signaling pathways (PTN, ANNEXIN, GDF, VISTA, CD6, ALCAM, SN) unique to sham, and three pathways (TNF, FASLG, LIFR) unique to AAA.

We next investigated the signaling changes in each population. Since SMC-1 and SMC-2 are enriched in transcripts related to the contractile and synthetic phenotypes, respectively (10), we examined signaling activities of these two cell populations more closely. AAA induction increased incoming SPP1 signaling and decreased incoming LAMININ signaling in both SMC-1 and SMC-2. In contrast, SMC-1 of the AAA group showed more outputs related to THBS signaling and less COLLAGEN signaling, whereas SMC-2 sent less THBS signaling and more COLLAGEN signaling upon AAA stimulation. Of note, SPP1 signaling in SMC-1 was AAA specific, which means in the sham group SPP1 signaling was undetectable (Figures 1E,F). All three macrophage populations sent out more SPP1 signaling and received less COLLAGEN signaling in AAA group compared to sham. Maph-2 also sent out more THBS signaling in the AAA group (Figures 1G–I). Fibroblasts showed elevated incoming COLLAGEN signals and reduced outgoing COLLAGEN signals (Supplementary Figure 1C).

### Cell-Cell Communication in Murine Peri-Adventitial Elastase Model

We next analyzed the scRNA-seq dataset published by Zhao et al. using the peri-adventitial elastase model (GSE152583) (11). In this model, infrarenal abdominal aortas from C57BL/6J mice were treated with 30 μl elastase or heat-inactivated elastase (control). Aortas were collected 7 or 14 days after elastase exposure or 14 days after heat-inactivated elastase exposure (control group) (11). After filtering out the red blood cells, we identified 16 cell populations using the markers from Zhao et al.'s study, including two fibroblast, two EC, three SMC, three macrophage, and two DC populations, as well as T cells, B cells, NK cells, and neural cells (Supplementary Figure 2A). Among the three macrophage populations, Maph-1 highly expressed the inflammatory gene *Il1b*, Maph-2 was enriched for the M2 macrophage marker *Cd163*, and Maph-3 expressed high levels of the proliferation marker Mki67 (Supplementary Figures 2D-G). SMC-1 expressed high level of contractile genes such as *Acta2* (Supplementary Figure 2H). SMC-2 highly expressed inflammatory genes such as *Neat1* and *Cebpb* (Supplementary Figures 2I,J).

**Figure 2:**
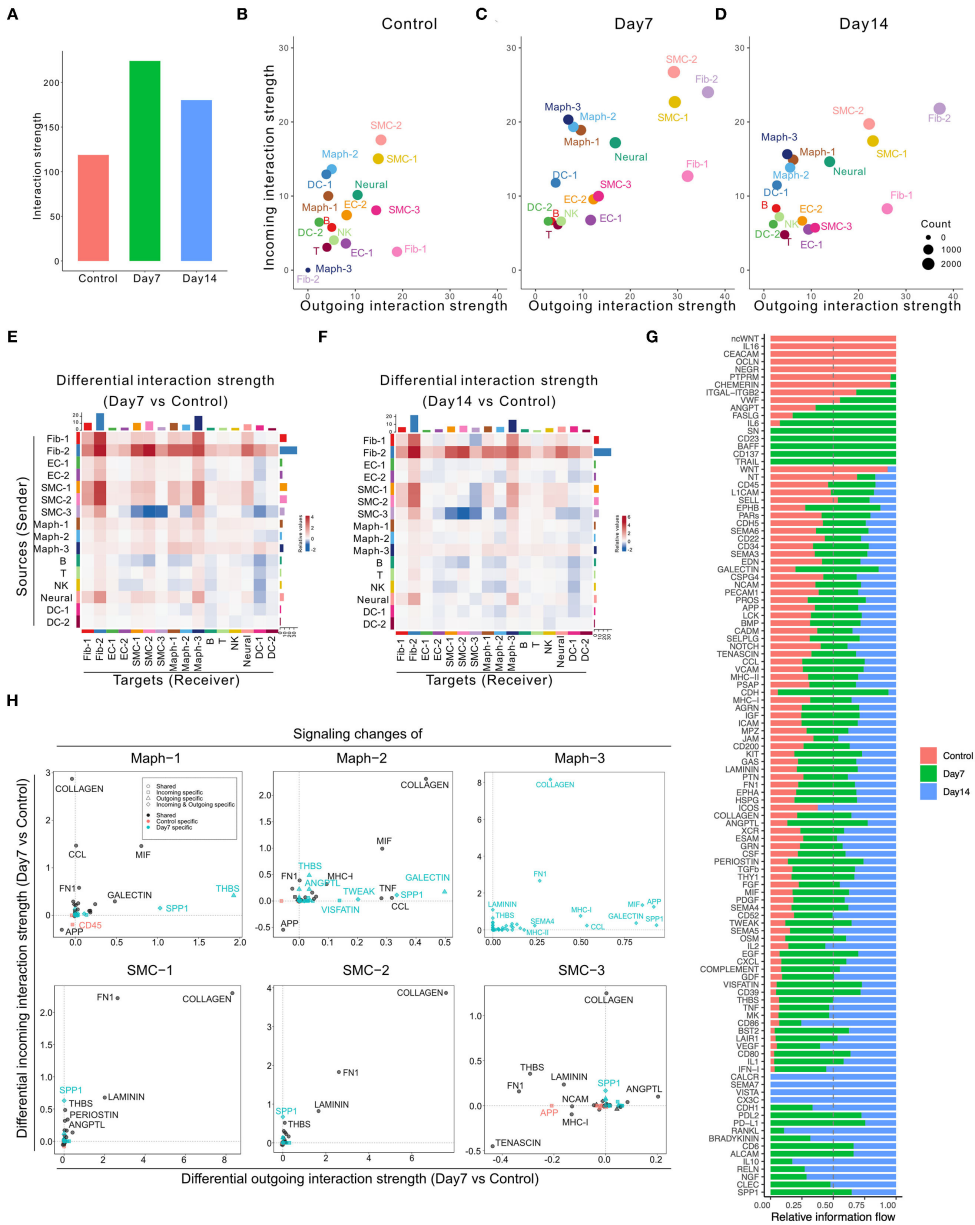
Inferred intercellular communication network in the murine perivascular elastase model. **(A)** Total interaction strength in control, Day 7, and 14 groups. **(B–D)** Scatter plot of incoming and outgoing interaction strength of each cell population in control **(B)**, Day 7 **(C)**, and Day 14 **(D)** groups. **(E,F)** Heatmap of differential interaction strength in Day 7 compared to control group **(E)**, and Day 14 compared to control group **(F)**. The top colored bar plot represents the sum of column of values displayed in the heatmap (incoming signaling). The right colored bar plot represents the sum of row of values (outgoing signaling). In the heatmap, red (or blue) represents increased (or decreased) signaling in Day 7 **(E)** or Day 14 **(F)** compared to control group. Relative value = the interaction strength from source to target in Day 7 **(E)** or Day 14 **(F)** group—the interaction strength from source to target in control group. **(G)** Overall information flow of each signaling pathway in control, Day 7, and 14 groups. Relative information flow is the ratio of the communication probability of a certain group (control, Day 7, or Day 14) relative to all groups combined. **(H)** Signaling changes of SMC and macrophage populations in Day 7 compared to control group.

Application of CellChat to this dataset identified 7,233 total interactions in the control group, 10,453 interactions in Day 7 group, and 9,343 interactions in Day 14 group (Supplementary Figure 2B). We also calculated the interaction strength of all cell populations in each group. The AAA induction by elastase treatment increased communication probability over the control group, with higher interaction strength at Day 7 than Day 14 (Figure 2A). Additionally, the AAA induction altered the communication patterns (Figures 2B–D). In both AAA groups, SMCs and fibroblasts served as the major signal source and target. Macrophage populations, especially Maph-3 and Maph-1, showed increased incoming signaling in elastase treatment groups compared to the control group (Figures 2B–D).

A more detailed dissection of the communication probability between each population highlights Fib-2 as an important node in aneurysmal tissues. Fib-2 received intensive signals from SMCs and fibroblasts and also sent abundant signals to SMCs and macrophages. Interestingly, SMC-3 became idle in response to elastase, sending fewer signals to the SMC populations compared to control (Figures 2E,F). Comparing Day 14 with Day 7, signals sent from Fib-2 to B cells and NK cells, as well as Fib-2 autocrine signaling were further elevated (Supplementary Figure 2C).

We next examined the overall changes in each signaling pathway in both conditions. As shown in Figure 2G and Supplementary Figures 2K,L, five signaling pathways were exclusively expressed in the control group (ncWNT, IL16, CEACAM, OCLN, and NEGR) and 21 pathways were only expressed by elastase treated groups. Among these 21 pathways, 5 of them were only expressed by Day 7 group (SN, CD23, BAFF, CD137, and TRAIL), and 4 out of 21 pathways were expressed only by Day 14 group (CALCR, SEMA7, VISTA, and CX3C).

We further evaluated the specific signaling pathways that were altered during the early aneurysmal response. In macrophages, particularly Maph-1 (the pro-inflammatory type), THBS signaling was prominent in the elastase treated group however absent in the control. All macrophage populations also showed elevated incoming COLLAGEN signaling and increased outgoing SPP1, MIF, and GALECTIN signaling. Outgoing COLLAGEN signaling was also upregulated, but only in Maph-2 and Maph-3. Similarly, COLLAGEN signaling was also the most increased incoming signaling pathway in all SMC populations and the most enhanced outgoing signaling pathway in SMC-1 and SMC-2 (Figure 2H). All fibroblast populations showed elevated incoming and outgoing COLLAGEN as well as FN1 signaling (Supplementary Figure 2M).

### Cell-Cell Communication in Murine Ang II Model

**Figure 3:**
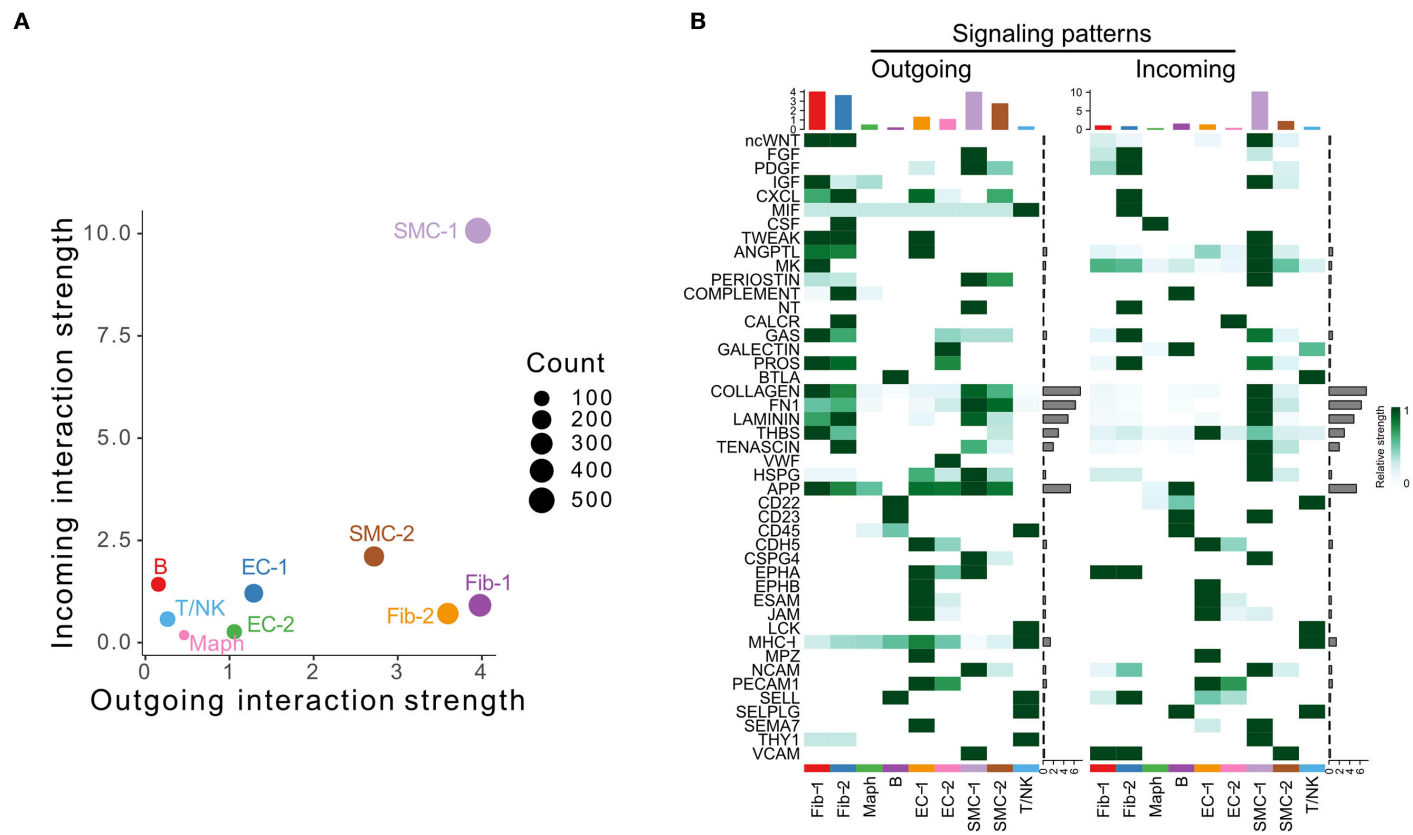
Inferred intercellular communication network in the murine Angiotensin II infusion model. **(A)** Scatter plot of incoming and outgoing interaction strength of each cell population in Angiotensin II group. **(B)** Outgoing and incoming signal strength of each signaling pathway in each cell population in Angiotensin II group.

We next examined the scRNA-seq dataset published by Hadi et al. (GSE118237) (3). In this model, *Apoe*^−/−^ mice were infused with 1,000 ng/kg/min Ang II *via* osmotic pumps for 28 days. No control group was included in this data set. Our analysis identified nine cell populations, including two SMC, two fibroblast, two EC populations, and macrophage, T/NK, and B cell populations (Supplementary Figure 3A). Specifically, SMC-1, the cell population characterized by enrichment of contractile marker Myh11 (Supplementary Figure 3B), was the major signal sender and receiver (Figure 3A). As shown in Figure 3B, fibroblast and SMC populations were the major signal source, and SMC-1 was the major signal receiver. COLLAGEN, FN1, LAMININ, THBS, APP, and TENASCIN were overall highly expressed signaling pathways.

### Cell-Cell Communication in Human Aneurysm Tissue

**Figure 4:**
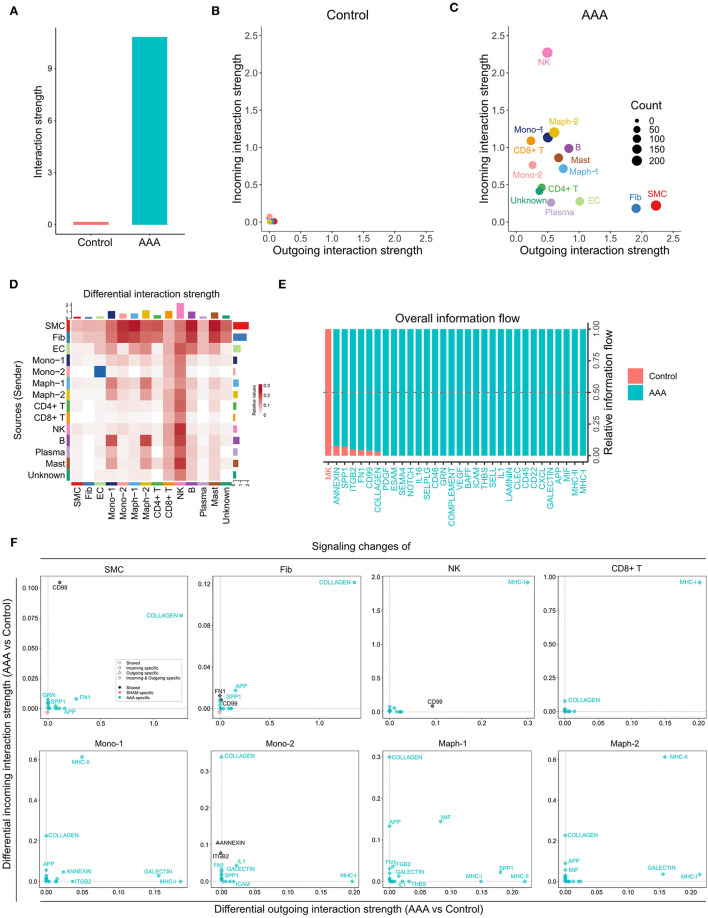
Inferred intercellular communication network in human AAA tissues. **(A)** Total interaction strength in control and AAA samples. **(B,C)** Scatter plot of incoming and outgoing interaction strength of each cell population in control **(B)** and AAA **(C)** groups. **(D)** Heatmap of differential interaction strength in AAA compared to control group. The top colored bar plot represents the sum of column of values displayed in the heatmap (incoming signaling). The right colored bar plot represents the sum of row of values (outgoing signaling). In the heatmap, red (or blue) represents increased (or decreased) signaling in AAA compared to control group. Relative value = the interaction strength from source to target in AAA group – the interaction strength from source to target in control group. **(E)** Overall information flow of each signaling pathway in control and AAA groups. Relative information flow is the ratio of the communication probability of a certain group (control or AAA group) relative to control and AAA combined. **(F)** Signaling changes of different cell populations in AAA compared to control group.

Davis et al. conducted scRNA-seq on infrarenal abdominal aortas of patients undergoing open aortic aneurysm repair (AAA group) or open aortobifemoral bypass (control group) (GSE166676) (9). We identified 14 populations in this dataset, including two monocyte and two macrophage populations, SMC, fibroblast, EC, CD4+ T cell, CD8+ T cell, NK, B, plasma, and mast cell populations, as well as one unknown population (Supplementary Figure 4A). We ran CellChat analysis on this dataset and identified 52 total interactions in the control group, and 972 total interactions in AAA group (Supplementary Figure 4B). The AAA group showed higher communication probability than the control group, as the interaction strength of control group was almost undetectable (Figures 4A,B). In the AAA group, SMC and fibroblast populations were the major signal senders, and the NK cell population was the major signal receiver (Figure 4C). Compared to the control group, the AAA group showed more signals sent from SMCs and fibroblasts to monocytes and macrophages, especially Mono-2 and Maph-1, as well as to B cells and mast cells. Signaling from Mono-2 to EC was the only decreased interaction in AAA compared to the control group (Figure 4D). Most signaling pathways were exclusively expressed in the AAA group, with only MK signaling being expressed primarily in the control group (Figure 4E). COLLAGEN signaling was enhanced at both the incoming and outgoing level in SMC and fibroblast populations, and was also elevated among incoming signals in monocytes, macrophages, CD4+ T cells, B cells, and mast cells, especially in Mono-2 and Maph-1. There were more incoming MHC-II signals in Mono-1 and Maph-2 populations, and increased outgoing MHC-II signals from Maph-1 and B cells (Figure 4F and Supplementary Figure 4C).

### Commonly Altered Signaling Pathways Among Different AAA Models

**Figure 5:**
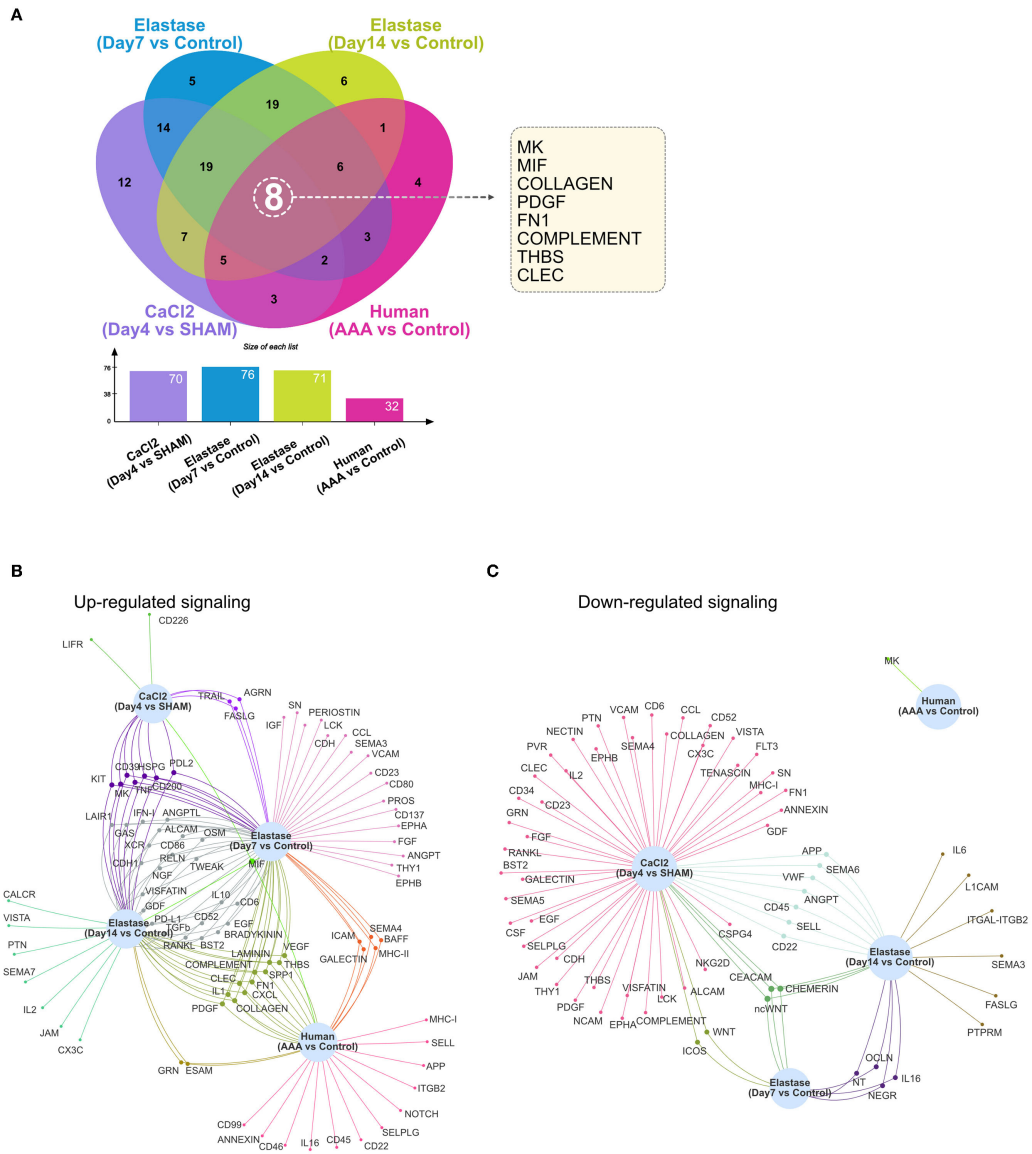
Altered signaling in murine and human AAA. **(A)** Venn diagram and bar graph of the numbers of altered signaling pathways in the murine CaCl_2_ model (CaCl_2_ treated compared to sham group), elastase model (Day 7 group compared to control group and Day 14 group compared to control group), and human AAA samples (AAA compared to control group). **(B,C)** Venn network of upregulated **(B)** or downregulated **(C)** signaling pathways in the murine CaCl_2_ model, elastase model, and human AAA samples.

As demonstrated in Figures 1–4, numerous signaling pathways were significantly altered in aneurysm tissues. Among the altered pathways (including both upregulated and downregulated pathways), eight were common to all murine models and time points as well as human AAA tissue. These include the MK, MIF, COLLAGEN, PDGF, FN1, COMPLEMENT, THBS, and CLEC signaling pathways (Figure 5A). MIF signaling was upregulated in all AAA groups compared to their respective controls, and eight signaling pathways were upregulated in all murine AAA groups (MIF, KIT, MK, CD39, HSPG, TNF, CD200, and PDL2) (Figure 5B). Regarding downregulated signaling pathways, three pathways were decreased in all murine AAA groups (ncWNT, CEACAM, CHEMERIN), while MK signaling was the only downregulated signaling pathway in human AAA (Figure 5C and Supplementary Figure 5).

### THBS Signaling in AAA

Since we have previously reported the importance of thrombospondin-1 (TSP1)—the matricellular protein encoded by *THBS1*—in two murine AAA models, we examined THBS signaling in a greater detail. Consistent with our previous reports (17, 18), THBS signaling was found to be common to the three murine scRNA-seq data sets analyzed in the current study as well as to human AAA (3, 9–11). However, in each murine model and human AAA, THBS signaling appeared to be produced by different cell populations, received by different cell populations, and the contribution of each ligand-receptor pair was different.

**Figure 6:**
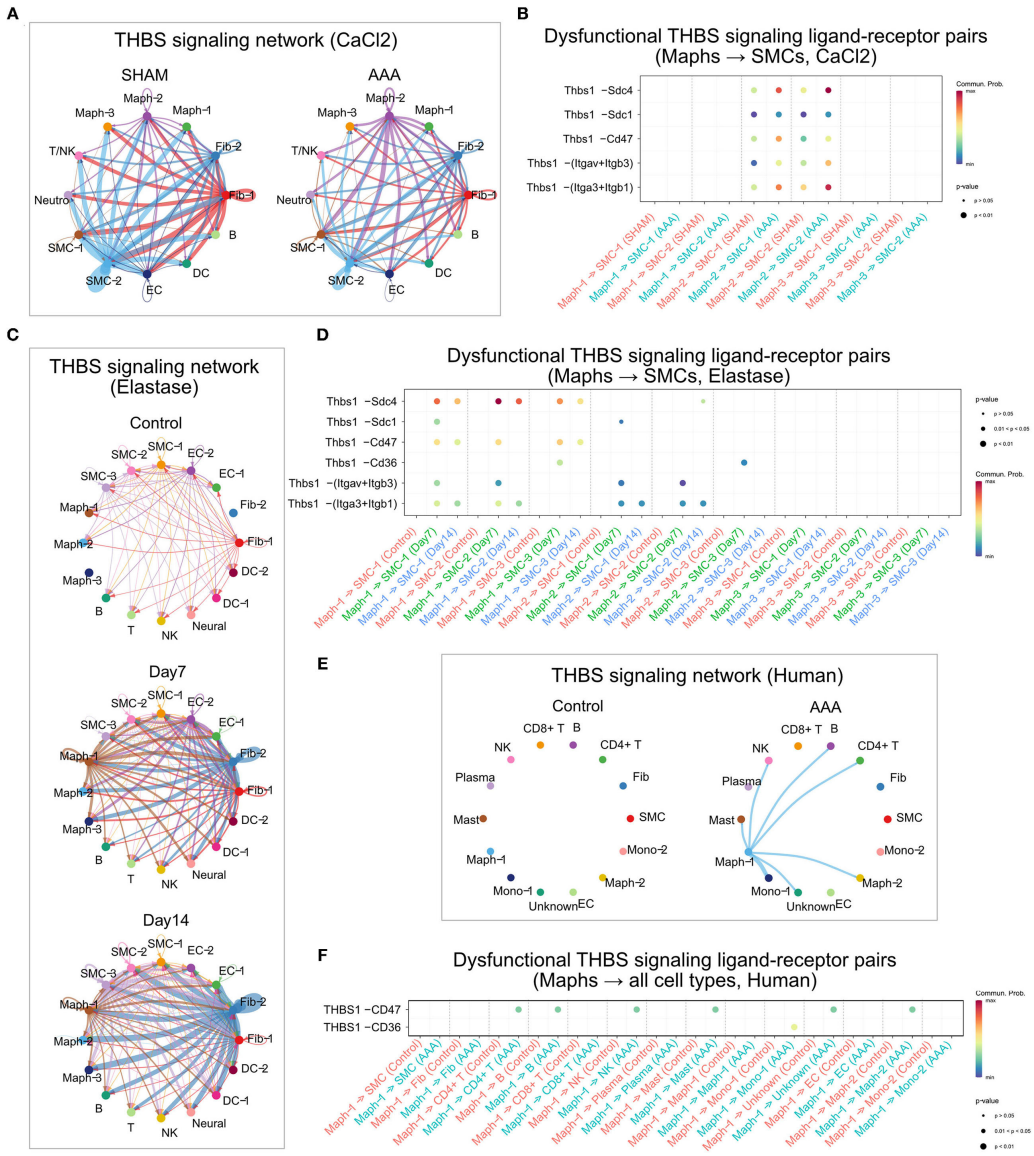
THBS signaling in murine and human AAA. **(A,C,E)** Circle plot of THBS signaling network in the murine CaCl_2_ model **(A)**, elastase model **(C)**, or human AAA **(E)**. The edge colors are consistent with the signal sender, and edge weights are proportional to the interaction strength. Thicker edge line indicates a stronger signal. **(B,D,F)** Bubble plot of the communication probability of all the significant ligand-receptor pairs that contributed to THBS signaling sent from macrophages to SMCs in the murine CaCl_2_ model **(B)** or elastase model **(D)**, and from Maph-1 to each cell population in human AAA **(F)**. The dot color and size represent the communication probability and *p*-values, respectively. *p*-values were computed from one-sided permutation test.

In the sham group of the CaCl_2_ model, SMC-2 and Fib-1 were the major cell types sending out THBS signaling. Upon AAA induction, THBS signaling sent from SMC-2 and Fib-1 populations was diminished, while signaling generated by Maph-2 was elevated. Specifically, THBS signaling sent from Maph-2 to SMC-1 or SMC-2 was most abundant in AAA (Figure 6A). Among the ligand-receptor pairs of THBS signaling, the Thbs1-Sdc4 ligand-receptor pair showed the highest communication probability, especially in Maph-2 to SMC-2 communication (Figure 6B). In contrast, the SMCs to macrophage communication that was prominent in sham tissue utilized the Thbs1-Cd47 ligand-receptor pair (Supplementary Figure 6A). As the communication probabilities were calculated based on the expression of ligands, receptors, and co-factors, we further plotted the gene expression of each ligand and receptor of THBS signaling. As shown in Supplementary Figure 6B, ligand Thbs1 was highly expressed by SMC-2 in the sham group, and reduced in AAA group. Thbs1 was also expressed by Maph-2, and its expression was elevated in AAA. Expression of receptor Sdc4 was also increased by AAA treatment in SMC-2, but Cd47 expression was comparable between sham and AAA in all populations.

In the peri-adventitia elastase model, THBS signaling was not highly expressed by the control group, but was elevated after elastase treatment. Signals from Fib-2 to SMCs and macrophages, and from Maph-1 to SMCs were increased the most by AAA induction (Figure 6C). Examination of each ligand-receptor pair in THBS signaling revealed that Thbs1-Sdc4 signaling was the dominant pathway from macrophages to SMCs, especially from Maph-1 to SMC-2 (Figure 6D). Thbs1-Sdc4 and Thbs1-Cd47 were responsible for communication from SMCs to macrophages, and from Fib-2 to fibroblasts, SMCs, and macrophages (Supplementary Figures 6C,D). Gene expression of each ligand and receptor of THBS signaling also showed that Thbs1 was strongly induced by AAA in Fib-2 and Maph-1, and that receptors Sdc4 and Cd47 were increased by AAA in Fib-2 and Maph-3 (Supplementary Figure 6E).

Similar to the elastase model, THBS signaling was not detected in control human aorta. In the human AAA group, THBS signaling originated from Maph-1 and was received by SMCs and other cell populations such as Mono-1, Maph-2, CD4+ T cells, B cells, NK cells, and Mast cells (Figure 6E). THBS1-CD47 was the main contributor of THBS signaling in the human AAA group (Figure 6F).

Since the murine Ang II model data set does not contain a control/sham group, we could only examine THBS signaling in the Ang II group. As shown in Supplementary Figures 6G,H, THBS signaling in this model was generated from Fib-1 and received by EC-1 (mediated by Comp-Cd36) and from Fib-2 to EC-1 (mediated by Thbs3-Cd36). Examination of ligand and receptor expression also confirmed that Comp and Thbs3 were highly expressed by Fib-1 and Fib-2 respectively, and that Cd36 was enriched in EC-1 (Supplementary Figure 6I).

## Discussion

Within a multicellular environment, cell-cell communication plays a fundamental role in governing tissue function, regulating individual cell processes, and intercellular relationships, thus driving tissue homeostasis and pathophysiology in states of health and disease (12, 13). Historically, studies investigating cell-cell communication could only be performed in the *in vitro* setting, examining one or two cell types and a limited number of genes at a time. This investigative approach fails to capture the rich network of cell-cell communications that occur in a diverse multicellular environment. In recent years, single-cell transcriptomics, which allows gene expression to be studied at the single-cell level, has generated an opportunity to examine complex networks of cell-cell communication in a multicellular community. Studies of single-cell transcriptomics in mouse models and human tissues have revealed cell clusters present in healthy and aneurysmal aortas. In this study, we inferred intercellular relationships between cell populations in AAA, in particular focusing on communications between SMCs and macrophages.

In an early-stage of the mouse CaCl_2_ model (Day 4 after AAA induction), we predicted that SMCs were actively sending and receiving signals in both sham and AAA groups. CaCl_2_ treatment increased signal sending from Maph-2 (pro-inflammatory macrophages) to SMC-1 (contractile SMCs). Among all the signaling pathways altered by AAA, SPP1 signaling was the most up-regulated outgoing signal in Maph-2, and the most elevated incoming signal in SMC-1. Similarly, in an early-stage of the elastase model (Day 7), macrophages also sent out more SPP1 signals compared to control, and increased incoming SPP1 signaling was detected in SMCs. Spp1 encodes osteopontin, which participates in vascular calcification and is associated with the synthetic SMC phenotype (19, 20). Our analysis suggests that pro-inflammatory macrophages may regulate SMC phenotypic changes through SPP1 signaling at an early-stage in murine AAA models.

In the perivascular elastase model, we inferred that fibroblasts became the primary signal source after elastase incubation. Signaling changes of each cell population also confirmed that COLLAGEN signaling was the most increased outgoing signaling in fibroblasts, and the most elevated incoming signaling in SMCs, macrophages, and endothelial cells in the Day 7 group compared to the control group. In contrast, SMCs, macrophages, and endothelial cells in the CaCl_2_ model received fewer COLLAGEN signals compared to the sham group; fibroblasts received more incoming COLLAGEN signals, but likely from synthetic SMCs. These results indicate that vascular remodeling and fibrosis may contribute to early progression of AAA in elastase model, but not CaCl_2_ model.

Our analysis revealed that THBS signaling was one of the eight signaling pathways that were altered in all AAA groups compared to their respective controls. Thrombospondins are a family of secreted glycoproteins that regulate multiple biological processes such as angiogenesis, apoptosis, and migration (21). Among the five family members, TSP1 is most studied in the context of AAA. How TSP1 contributes to AAA pathogenesis is still not entirely clear. Our group found that TSP1 level was upregulated in human AAA as well as in murine models including CaCl_2_, Ang II, and intraluminal elastase perfusion model (17). In contrast, Krishna et al. reported reduced TSP1 expression in aneurysm tissues from AAA patients (22). Similarly, opposing outcomes were observed when globally deleting Thbs1 in mouse models for AAA (17, 22). These controversial findings may highlight the cell type specificity of TSP1 functions in aneurysmal disease. TSP1 binds to a wide range of receptors including syndecans, CD36, integrins, and CD47 (23, 24), but the role of these ligand-receptor pairs in AAA has not been investigated.

In this study, we inferred that synthetic SMCs (SMC-2) were the main source of THBS signaling in the sham group of CaCl_2_ model. AAA treatment decreased THBS signals sent from SMC-2, and enhanced THBS signals sent from pro-inflammatory macrophages (Maph-2) to other cell types, especially SMCs. This finding is consistent with our previous publication in which we showed that macrophages are the major source of TSP1 in murine CaCl_2_, Ang II models, and human AAA tissues (18). By analyzing each ligand-receptor pair of THBS signaling between macrophages and SMCs, we identified that Thbs1-Sdc4 was the most elevated pathway sent from Maph-2 to SMCs (especially synthetic SMC-2), and that Thbs1-Cd47 signaling sent from SMC-2 to macrophages (especially pro-inflammatory Maph-2) was decreased by AAA. Expression of each THBS signaling gene further showed that ligand Thbs1 expression was increased in Maph-2 and decreased in SMC-2. Receptor Sdc4 expression was elevated in SMC-2, explaining the signaling changes between macrophages and SMCs.

In the elastase model, THBS signaling was relatively quiescent in the control group, but was induced by elastase stimulation. Similar to the CaCl_2_ model, the Thbs1-Sdc4 pathway was also the most increased pathway sent from pro-inflammatory macrophages (Maph-1) to SMCs (especially synthetic SMC-2). Of note, Thbs1-Sdc4 signaling was increased the most at Day 7, and slightly decreased at Day 14. Expression of each THBS signaling gene also showed that, in Maph-1, Thbs1 was robustly increased at Day 7 and slightly decreased at Day 14 compared to control. Thbs1-Sdc4 and Thbs1-Cd47 were the major signaling pathways sent from SMCs (SMC-3) to macrophages. In contrast to observations in macrophages, these two signaling pathways were most elevated at Day 14, consistent with the up-regulated expression of Thbs1 in SMC-3 at Day 14. These results suggested that THBS signaling from pro-inflammatory macrophages to SMCs was activated at early stage, and from SMCs to macrophages at a later stage.

This study has several limitations. First, our analysis infers cell-cell communication based on gene expression of ligands and their receptors and cofactors, while cell signaling ultimately occurs at the protein level. In the setting of post-transcriptional and post-translational modifications, as well as multi-subunit protein complex assembly, gene expression may not always accurately reflect protein level. Second, the proximity of cells, ligands, cofactors, and receptors to one another is critically important to cell signaling. Many ligands activate signaling cascades either by diffusing through the extracellular environment from a sender cell to a nearby receiver cell, or through gap-junctions between directly adjacent cells. Unfortunately, this spatial information is not captured in scRNA-seq data (12). In addition, the ligand-receptor database used in this study is CellChatDB, which is included in the CellChat package. It is a manually curated database of literature-supported ligand-receptor interactions in both human and mouse. CellChatDB in mouse contains 2,021 validated molecular interactions, and is composed of 60% secreted autocrine/paracrine signaling interactions, 21% extracellular matrix (ECM)-receptor interactions, and 19% cell-cell contact interactions. CellChatDB in human contains 1,939 validated molecular interactions, and is composed of 61.8% paracrine/autocrine signaling interactions, 21.7% extracellular matrix (ECM)-receptor interactions, and 16.5% cell-cell contact interactions (14). The inference of cell-cell communication relies highly upon the quality of the ligand-receptor database, and different ligand-receptor databases used in different computational tools could lead to various predicted results. Finally, currently there is no single animal model that mimics the full clinical characteristics of human AAA, and human AAA tissue can only be obtained at an advanced stage during surgical repair. In this study, we predicted cell-cell communication in human AAA and different animal models at different disease stages. Validation of these intercellular signaling networks would be informative.

In conclusion, we inferred intercellular communication networks in the murine CaCl_2_ model, elastase model, and Ang II model, as well as in human AAA. Our analysis also predicted commonly altered signaling pathways in AAA, paying particular attention to THBS signaling between different cell populations. Our data provide a guide for future experimental investigations to elucidate the cell-cell communications driving AAA.

## Data Availability Statement

The datasets presented in this study can be found in online repositories. The names of the repository/repositories and accession number(s) can be found in the article/Supplementary Material.

## Author Contributions

HY, TZ, and BL designed research studies. HY analyzed data. TZ, ED, and BL wrote the manuscript. All authors contributed to the article and approved the submitted version.

## Funding

This study was supported by the National Institute of Health (R01HL149404 and R01HL158073-01 to BL, and F32HL158171-01 to ED) and the American Heart Association (17POST33680095 and 20CDA35350009 to TZ).

## Conflict of Interest

The authors declare that the research was conducted in the absence of any commercial or financial relationships that could be construed as a potential conflict of interest.

## Publisher's Note

All claims expressed in this article are solely those of the authors and do not necessarily represent those of their affiliated organizations, or those of the publisher, the editors and the reviewers. Any product that may be evaluated in this article, or claim that may be made by its manufacturer, is not guaranteed or endorsed by the publisher.
