# Supplementary material for: Deciphering Cell-Cell Communication in Abdominal Aortic Aneurysm From Single-Cell RNA Transcriptomic Data
Source: Front Cardiovasc Med. 2022 Feb 4;9:831789. doi: 10.3389/fcvm.2022.831789 (PMC8854649; doi:10.3389/fcvm.2022.831789)
Supplement: Supplementary file 1 [file Data_Sheet_1.pdf]

Supplemental Figure 1

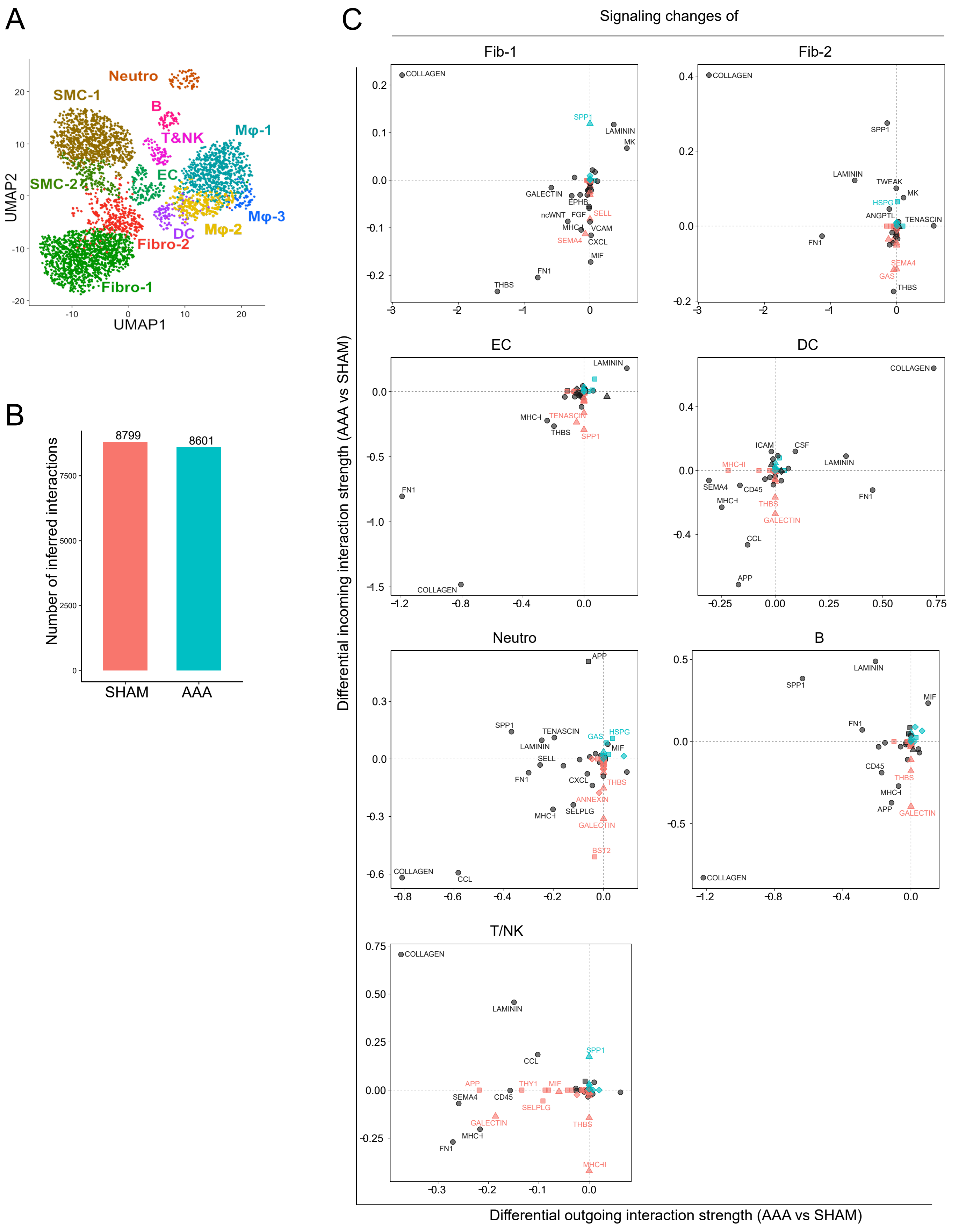

Supplemental Figure 1. Signaling pattern in the $CaCl_2$-induced murine abdominal aortic aneurysm (AAA) model. (A) Uniform manifold approximation and projection (UMAP) plot of cell populations presented in sham and AAA combined. (B) Number of inferred interactions in sham and AAA groups. (C) Signaling changes of different cell populations in AAA compared to sham group.

Supplemental Figure 2

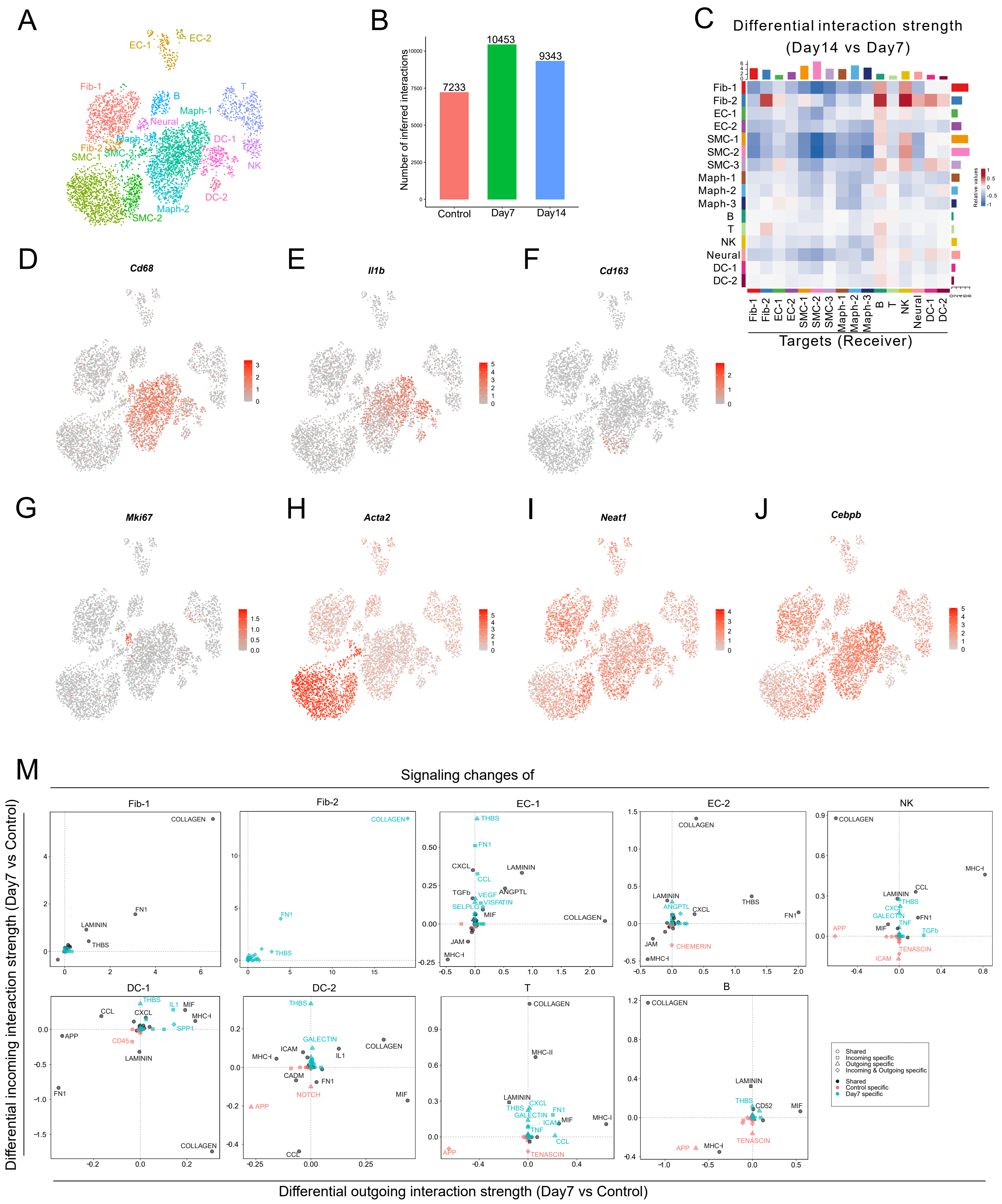

(To be continued)

Supplemental Figure 2 (continued)

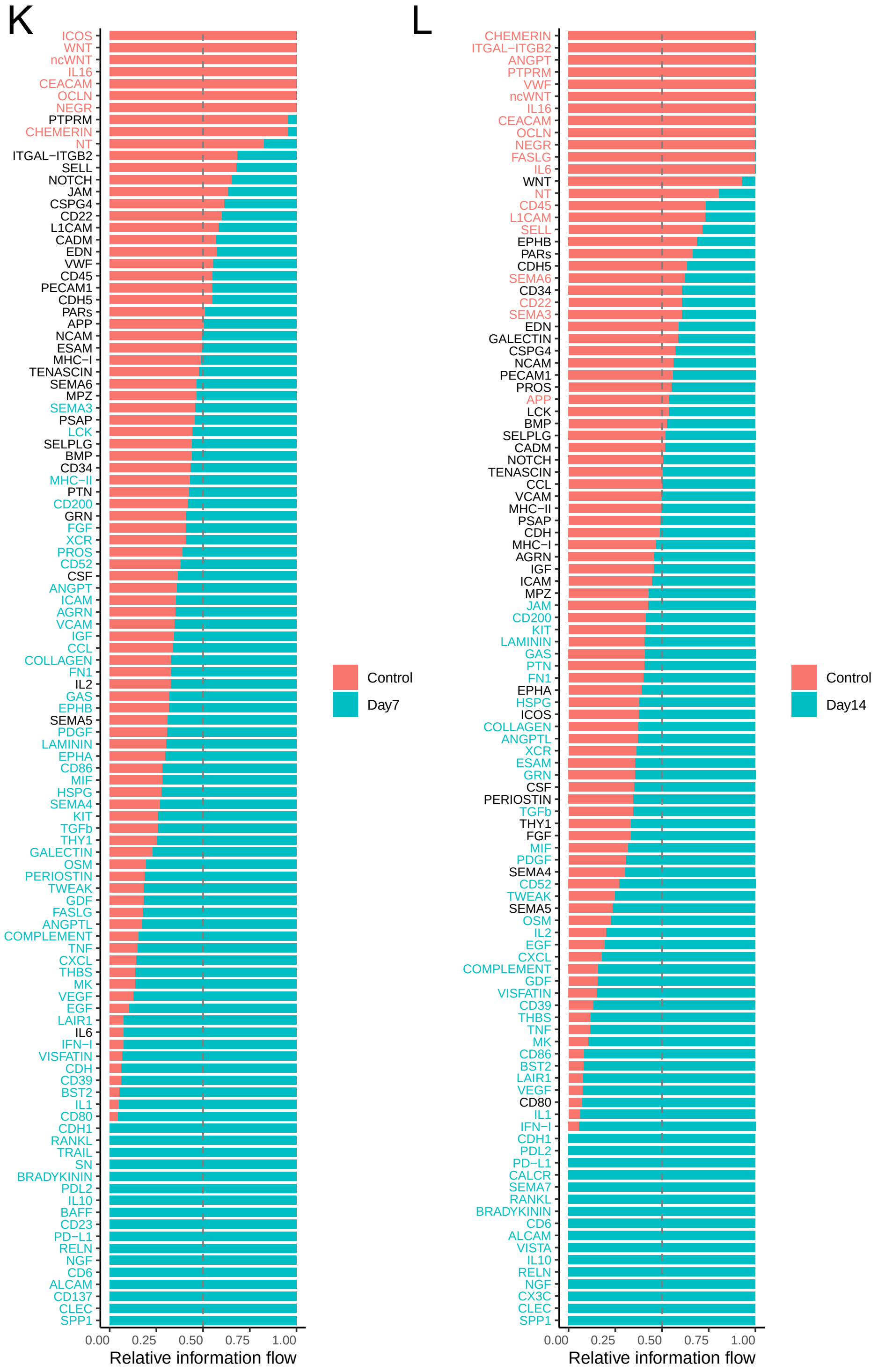

Supplemental Figure 2. Signaling profile in the murine perivascular elastase model. (A) Cell populations present in control, Day 7, and Day 14 combined. (B) Number of inferred interactions in control, Day 7, and Day 14 groups. (C) Heatmap of differential interaction strength in Day 14 compared to Day 7 group. The top colored bar plot represents the sum of column of values displayed in the heatmap (incoming signaling). The right colored bar plot represents the sum of row of values (outgoing signaling). In the heatmap, red (or blue) represents increased (or decreased) signaling in Day 14 compared to Day 7 group. Relative value = the interaction strength from source to target in Day 14 group - the interaction strength from source to target in Day 7 group. (D-J) Distribution of marker genes in control, Day 7, and Day 14 combined. (K&L) Information flow of each signaling pathway in control and Day 7 groups (K) or in control and Day 14 (L) groups. Relative information flow is the ratio of the communication probability of a certain group [control or Day 7 in (K), control or Day 14 in (L)] relative to control and Day 7 combined (K) or control and Day 14 combined (L). (M) Signaling changes of different cell populations in Day 7 compared to control group.

Supplemental Figure 3

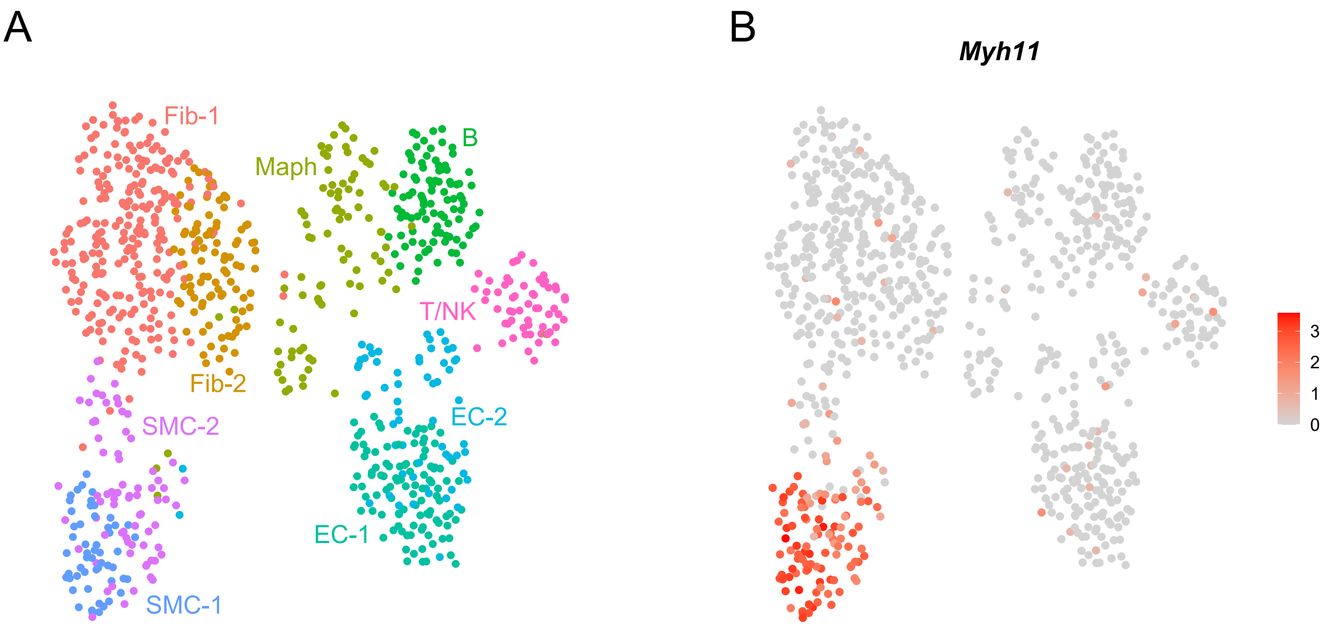

Supplemental Figure 3. Cell populations in the murine Angiotensin II model. (A) Cell populations present in Angiotensin II group. (B) Distribution of SMC marker Myh11 in Angiotensin II group.

Supplemental Figure 4

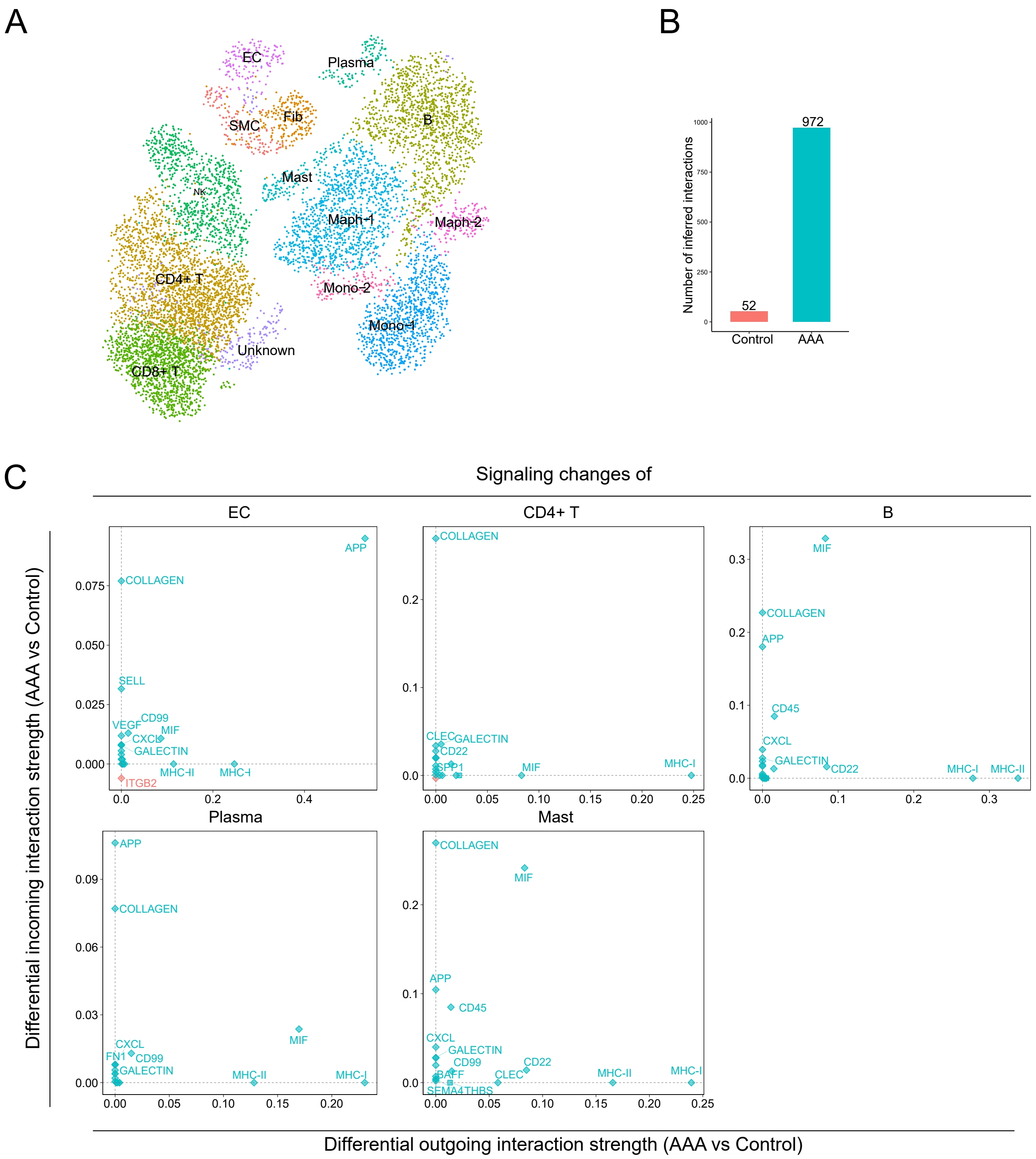

Supplemental Figure 4. Transcriptomic profile of human AAA sample. (A) Cell populations in human control and AAA groups combined. (B) Number of inferred interactions in control and AAA groups. (C) Signaling changes of different cell populations in AAA compared to control group.

Supplemental Figure 5

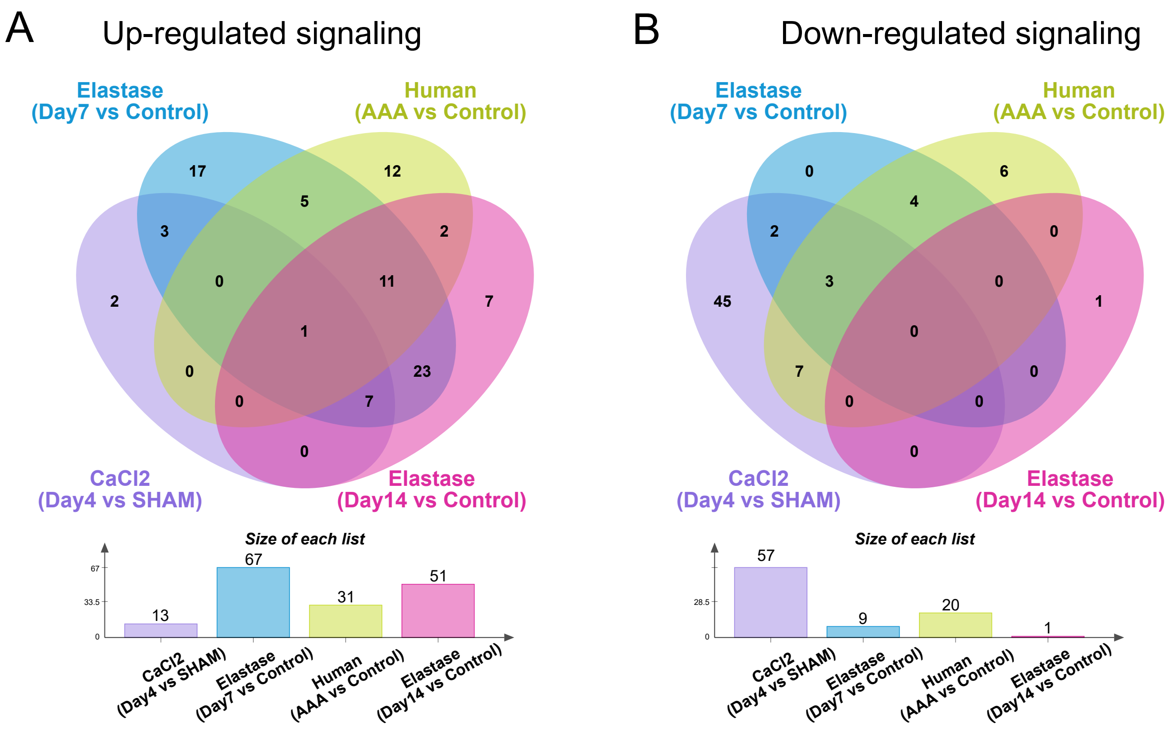

Supplemental Figure 5. Altered signaling in murine and human AAA. Venn diagram and bar graph of the numbers of up-regulated (A) or down-regulated (B) signaling pathways in the murine $CaCl_2$ model ($CaCl_2$ treated compared to sham group), elastase model (Day 7 group compared to control group and Day 14 group compared to control group), and human AAA samples (AAA compared to control group).

Supplemental Figure 6

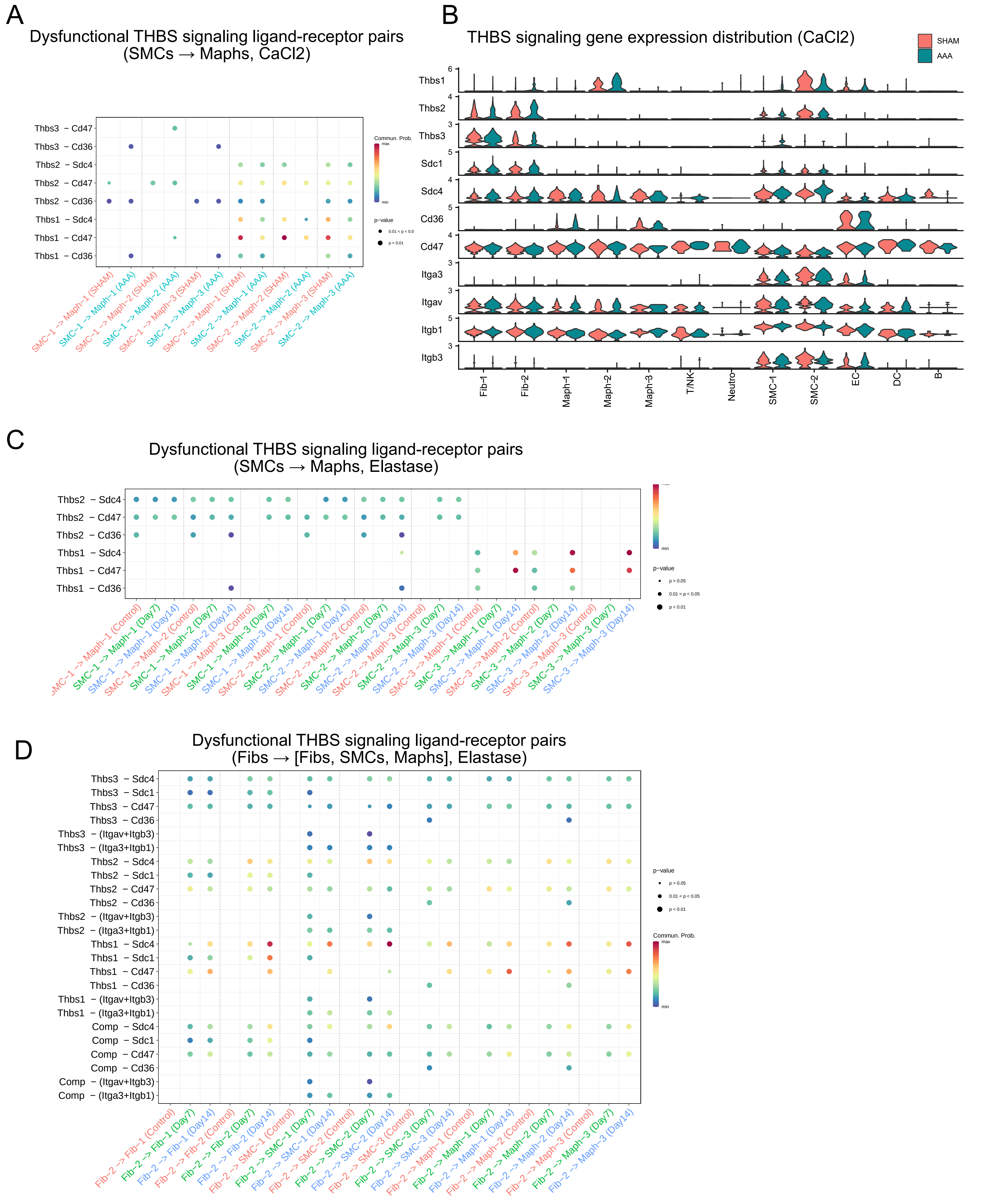

(To be continued)

Supplemental Figure 6 (continued)

E

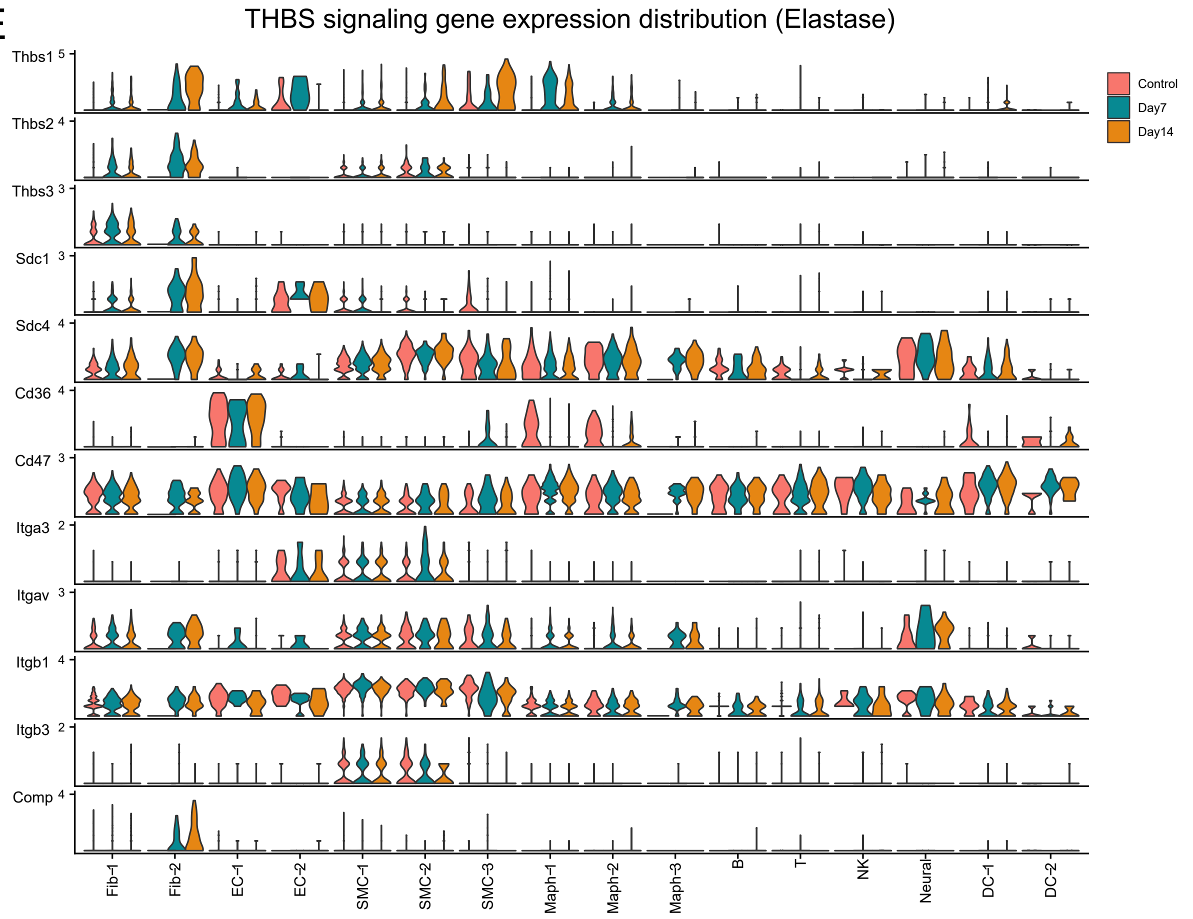

F

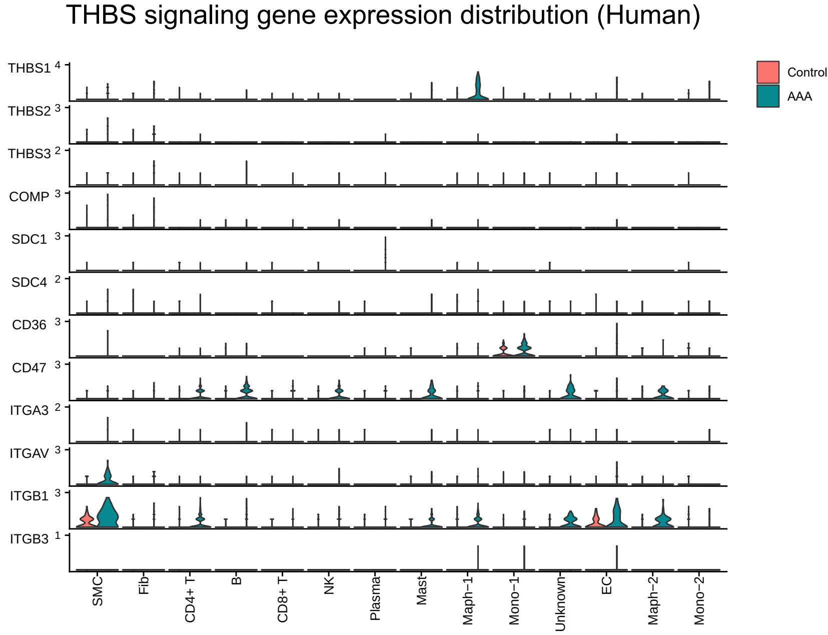

G

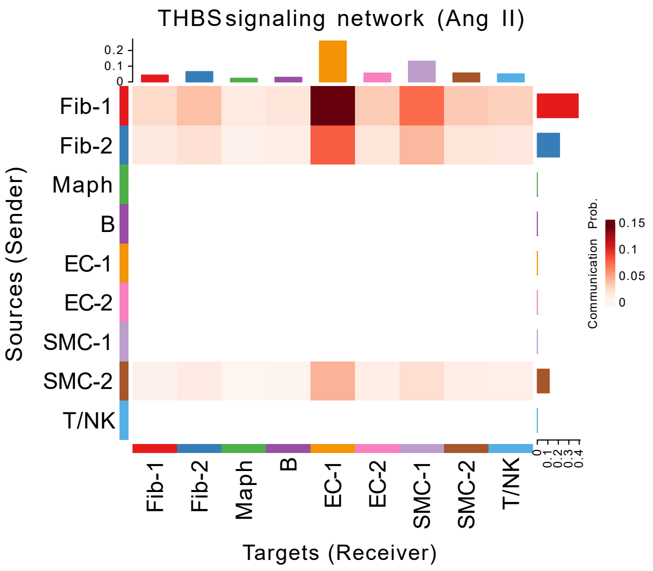

(To be continued)

Supplemental Figure 6 (continued)

H

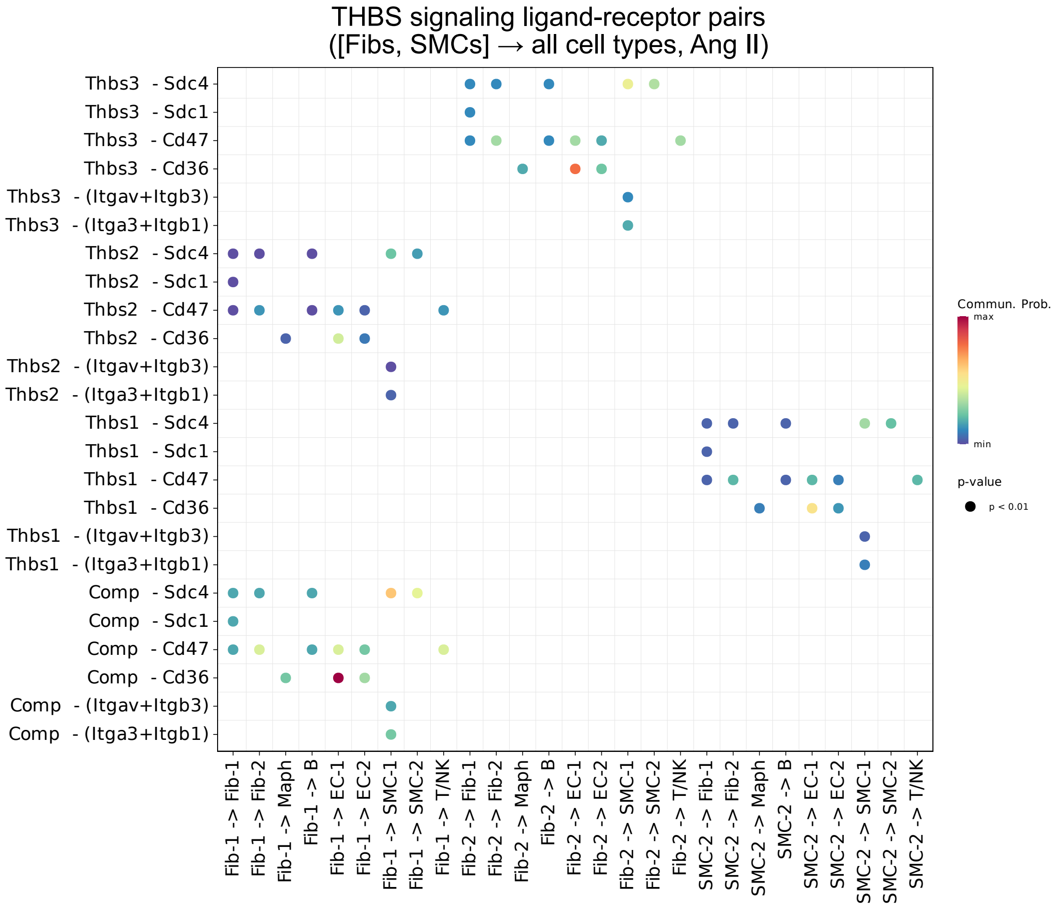

I

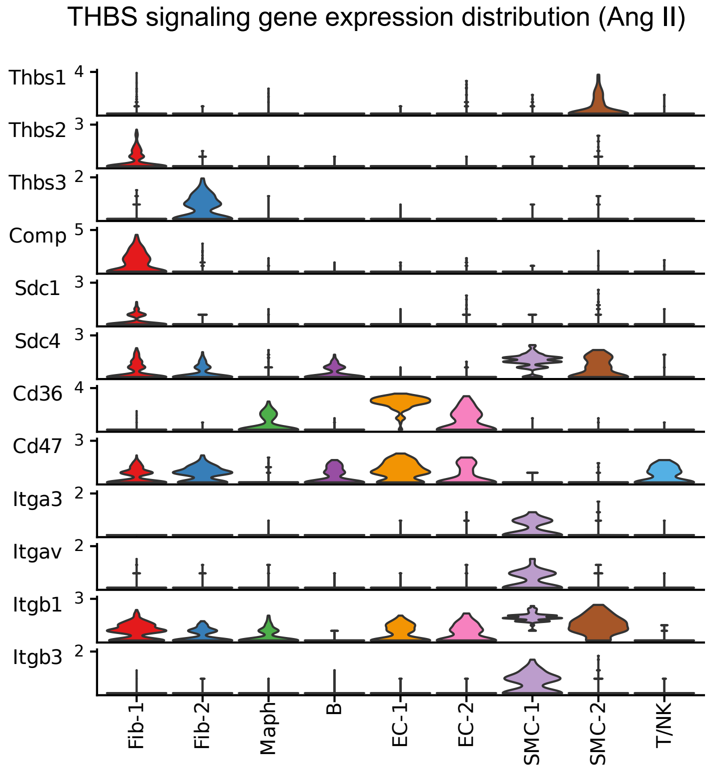

Supplemental Figure 6. THBS signaling ligand-receptor pairs and gene distribution in murine and human AAA. (A, C, D, H) Bubble plot of the communication probability of all the significant ligand-receptor pairs that contributed to THBS signaling sent from SMCs to macrophages in the murine $CaCl_2$ model (A) or elastase model (C), from fibroblasts to indicated cell types in elastase model (D), and from fibroblasts and SMCs to all cell populations in Angiotensin II model (H). The dot color and size represent the communication probability and p-values, respectively. p-values were computed from one-sided permutation test. (B, E, F, I) Gene expression distribution of THBS signaling genes in the murine $CaCl_2$ model (B), elastase model (E), human AAA (F), and the murine Angiotensin II model (I). The gene expression value labeled in the vertical axis is the log1p of normalized unique molecular identifier (UMI) counts.
